# Chemogenetic stimulation of the infralimbic cortex reverses alcohol-induced fear memory overgeneralization

**DOI:** 10.1038/s41598-019-43159-w

**Published:** 2019-04-30

**Authors:** M. J. Scarlata, S. H. Lee, D. Lee, S. E. Kandigian, A. J. Hiller, J. G. Dishart, G. E. Mintz, Z. Wang, G. I. Coste, A. L. Mousley, I. Soler, K. Lawson, A. J. Ng, J. L. Bezek, H. C. Bergstrom

**Affiliations:** Vassar College, Department of Psychological Science, Program in Neuroscience and Behavior, Poughkeepsie, NY 12604 USA

**Keywords:** Classical conditioning, Prefrontal cortex, Stress and resilience, Amygdala

## Abstract

Post-traumatic stress disorder (PTSD) and alcohol use disorder (AUD) are often comorbid. Drinking tends to increase following trauma, which may exacerbate PTSD-related symptoms. Despite a clear relationship between excessive alcohol use and PTSD, how alcohol impacts the expression of traumatic fear remains unclear. This study aims to determine the neurobehavioral impact of chronic alcohol (ethanol; EtOH) on the expression of established fear memories in C57BL/6 N mice. We show that chronic EtOH selectively augments cued fear memory generalization and impairs fear extinction retrieval, leaving the expression of the original cued response intact. Immunohistochemistry for Arc/arg3.1 (Arc) revealed EtOH-induced decreases in Arc expression in the infralimbic cortex (IL) and basolateral amygdala complex (BLA) that were associated with cued fear memory overgeneralization. Chemogenetic stimulation of IL pyramidal neurons reversed EtOH-driven fear memory overgeneralization, identifying a role for the IL in cued fear memory precision. Considering the modulatory influence of the IL over conditioned fear expression, these data suggest a model whereby chronic EtOH-driven neuroadaptations in the IL promote fear memory overgeneralization. These findings provide new mechanistic insight into how excessive alcohol use, following exposure to a traumatic event, can exacerbate symptoms of traumatic fear.

## Introduction

In those that develop post-traumatic stress disorder (PTSD), there is greater risk of developing an alcohol use disorder (AUD)^1,2^. Alcohol use tends to increase following a traumatic experience^3–5^. Importantly, in those with comorbid AUD and PTSD, the severity of trauma-related symptoms can be worse^6^, which may lead to poorer treatment outcomes^1,7^, especially among veterans^8^. Despite the clear relationship between excessive alcohol use and PTSD symptomatology, the mechanistic interactions remain unknown.

PTSD is characterized by exaggerated and situationally inappropriate defensive responses to perceived threat^9^. Threat/aversive/avoidance (“fear”) conditioning is a leading model for studying the neurobehavioral mechanisms of threat learning and memory^10–12^. In fear conditioning, an initially neutral sensory stimulus gains the ability to evoke conditioned defensive responses (conditioned response; CR) after being paired with a naturally aversive unconditioned stimulus (US)^13^. In fear extinction, the conditioned response (CR) is gradually reduced when the conditioned stimulus (CS) is repeatedly presented, in the absence of the US. Rodent models are advantageous for studying directional interactions between alcohol (ethanol; EtOH) and fear conditioning because EtOH can be precisely delivered, timed, and isolated to a circumscribed learning and memory phase. In most prior studies modeling the relationship between chronic EtOH exposure and fear conditioning, EtOH exposure was timed prior to learning. Therefore, these studies tested the effects of EtOH on *both* learning and retrieval. The results of these studies are equivocal, with evidence for impairment, enhancement, or no effect of EtOH on subsequent retrieval of the conditioned fear memories^14–21^. While only a handful of studies have investigated the impact of EtOH on the expression of established fear memories^22–25^, no studies to date have addressed how EtOH might influence the expression of an established fear extinction memory.

This study aims to investigate the neurobehavioral impact of chronic EtOH on the expression of previously established fear and extinction memories in C57BL/6 N mice. Our experimental approach enables the parsing of EtOH’s selective impact on mechanisms underlying fear retrieval, versus learning. In line with previous findings^17,22^, we hypothesized that chronic EtOH after fear conditioning and extinction would augment conditioned responding. In the first study, chronic EtOH exposure was isolated to a temporal window following the consolidation of auditory cued fear conditioning or extinction. Several days following EtOH exposure, memory retrieval was tested for the original CS, for an alternate tone frequency (generalization test), or for extinction retrieval. The study of fear memory retrieval, and generalization and extinction processes in particular, has considerable translational relevance since PTSD has been linked with both overgeneralization and impaired extinction of traumatic fear^26–29^. In the second study, EtOH-induced neuroadaptations following fear memory retrieval were identified by mapping the expression of the activity-regulated cytoskeletal Arc/arg3.1 protein in substructures of the medial prefrontal cortex (mPFC) and basolateral amygdala complex (BLA) using immunohistochemistry. Arc protein has been validated as a cellular marker of synaptic plasticity underlying learning and memory^30^, including Pavlovian fear conditioning^31,32^. In the third study, to determine whether modulating mPFC signaling normalizes EtOH-induced changes in fear memory expression, a Gq-coupled, cell-type selective, Designer Receptors Exclusively Activated by Designer Drug (DREADD) system was used to excite principal neurons (CaMKIIa-hM3D-Gq) in the IL during cued fear memory retrieval.

## Materials and Methods

### Animals

Adult (70–207 days old, median = 140 days old; weight 24.3–47.1 g, median weight = 34.6 g), male C57BL/6 N (B6) mice were used in all experiments. Mice derived from a common stock (Charles River Laboratory, Kingston, NY) and were bred at Vassar College over multiple generations. Mice were group-housed (at least 2/cage) in standard cages (minimal enrichment) in a temperature (20 °C), humidity (65%), and 12 hr light/dark cycle (lights on 0600) controlled vivarium. Food and water were available *ad libitum* and cages were changed 2 times/week. All experimental procedures were conducted in accordance with the National Institutes of Health guidelines on the Care and Use of Animals in Research and approved by the Vassar College Institutional Use and Animal Care Committee (IACUC). Disclosure of animal housing, husbandry, and experimental procedures follow principles for transparent reporting and reproducibility in behavioral neuroscience^33,34^.

### Fear conditioning

All experiments were conducted during the light cycle. All training and testing followed a 30 min habituation period in one of two adjacent holding rooms. Mice underwent fear conditioning using identical procedures across all experiments. Fear conditioning was conducted in unaltered commercial chambers (20 × 30 × 18 cm) located within sound-attenuating cabinets (58 × 61 × 45 cm) using Graphic State software for controlling and delivering the tone and foot shock stimuli (Coulbourn Instruments, Holliston, MA USA). Prior to all training and testing, the decibel level for the auditory tone frequency was measured in each chamber using a sound level meter (R8050, REED Instruments, Wilmington, NC USA) and calibrated to 73–75 dB (background dB = 67–69). Mice were placed in the fear conditioning chamber for 180 sec prior to three pairings of an auditory tone CS (20 sec, 5-kHz, 75 dB) that co-terminated with an electric foot shock US (0.5 sec, 0.6 mA). The CS/US pairings were separated by variable inter-trial intervals (ITI) (20 and 80 sec). Mice were removed from the chamber 60 sec after the final CS/US pairing. The total training time was 400 sec. The chambers were thoroughly cleaned with a 70% EtOH solution between mice.

### EtOH exposure

EtOH exposure procedures were identical across all experiments. All mice were randomly assigned to either the EtOH or control groups following training. A 20% v/v solution of ethanol (EtOH) was prepared fresh weekly by diluting 95% EtOH stock solution (Pharmco Aaper, Brookfield, CT USA) with either sterile physiological (0.9%) saline or dH_2_0. Mice received intraperitoneal (i.p.) injections of EtOH (2.5 g/kg) or the equivalent volume of saline (control) once daily over 5 consecutive days in the home cage. The EtOH volume administered was based on daily recorded body weight at a volume of 0.01 mL/g (0.53 ± 0.01 mL per injection). A 26-gauge, 0.37-inch length needle was used for all injections. All injections were administered during the light cycle between 0800 and 1200. The location of the i.p. injection was alternated each day across the midline to minimize skin irritation. The EtOH dose (2.5 g/kg) chosen for these experiments was shown to produce blood ethanol concentration (BEC) levels of 1.87 mg/mL in adult male C57BL/6 mice when tested 15 min following i.p. injection^35^. Similarly, binge-like drinking levels, using “drinking-in-the-dark” procedures, have been shown to produce BECs > 1.0 mg/mL over a 3 hr period in mice^36^. Importantly, 2.5 g/kg EtOH i.p. injections have previously been shown to change rodent brain structure and function^35,37–40^.

### Cued fear memory test

To isolate CS-elicited (cued) freezing from contextual (background) freezing (definition for “freezing” detailed below), the training context (Context A) was disguised for testing (Context B) using the following procedures: (1) mice were transported from the vivarium to the holding room prior to testing using distinctive cages, carts, and covering, (2) the lighting and ambient background noise of the holding and testing rooms were also changed by varying the illuminance and using a fan, (3) the tactile features of the testing chamber were changed by covering the shock bars with white plexiglass and a loose, thin layer of clean bedding, (4) the visual features of the testing chamber were disguised by covering the walls with distinct black- and white-striping, (5) the testing chambers were thoroughly cleaned between mice using a 1% acetic acid solution.

### Cued fear memory generalization

One day following fear conditioning, mice were placed into the training and testing contexts on consecutive days for 30 min each. Pre-exposure to the testing context was included in this experimental design to reduce baseline freezing to context shift^41^. The next day, mice were exposed to EtOH (2.5 g/kg, i.p.) or control once daily over 5 consecutive days. Following three EtOH-free days in the home cage, mice were tested for cued fear memory discrimination and generalization using a novel 3-kHz “non-target” tone at the same intensity (75 dB) and duration (20 sec) as the original CS. The novel tone was presented 50 times with a 5 sec ITI. This CS frequency was chosen based on previous work showing that B6 mice can discriminate a 3-kHz frequency tone one day following training at 5-kHz^42^. A separate cohort of mice was fear conditioned and then exposed to EtOH using identical procedures as those described above but presented with the “target” CS (5-kHz, 75 dB, 20 sec) for comparison. Ten days following testing (Remote test), mice were placed back into context B and presented with the same 3 tones (3-kHz or 5-kHz frequency) and variable ITI (20 and 80 sec).

### Cued fear extinction

The day following fear conditioning, mice were returned to the training context for 24 min and 45 sec (context A extinction). The next day, mice underwent cued fear extinction training in the novel testing context. Following 180 sec, mice were presented with 50 temporally massed (5 s ITI) CS presentations (20 sec, 5-kHz, 75 dB)^43^. The total training time was 24 min and 45 sec. The next day, mice were exposed to EtOH on five consecutive days using procedures outlined above. After three EtOH-free days in the home cage, mice were placed back into the testing context for an extinction memory test. Following 180 sec, mice were replayed the CS (20 sec, 5-kHz, 75 dB) three times separated by a variable ITI (80 and 20 sec). Context renewal was tested the following day in a subset of mice placed in the training context for 180 sec followed by the presentation of three CSs (20 sec, 5 kHz, 75 dB) and variable ITI (20 and 80 sec). To test the lasting effects of EtOH on fear extinction memory (remote test), mice were placed back into testing context 15 days following EtOH exposure and were replayed three CSs (20 sec, 5-kHz, 75 dB) with variable ITI (80 and 20 sec). For all testing procedures, mice were removed from the chamber 60 sec following the final CS presentation.

### Elevated plus maze

Approximately 4 hours following the cued fear memory generalization test, a subset of mice (n = 11–13/group) were placed into the elevated plus maze (EPM) for 10 min. The EPM was elevated 64 cm above the floor with 53 cm arm length, 7 cm arm width and 35 cm wall height. The EPM was thoroughly cleaned with a 70% EtOH solution between mice. The number of entries and duration in each arm, immobility and total distance travelled were recorded by digital camera and analyzed offline using SMART tracking software.

### Novel open field

A separate cohort of mice (n = 5–7/group) were administered EtOH (2.5 g/kg, i.p.) or control once daily over 5 consecutive days using identical procedures to those described above. After three EtOH-free days in the home cage, mice were placed in the lower left corner of a novel open field apparatus (54 × 39 cm) and allowed to freely explore for 10 min. Importantly, the time frame between EtOH exposure and testing in the novel open field was identical to the time frame used in the fear generalization experiments (4 days). The duration and distance traveled in the periphery and center of the open field (18 × 13 cm) was quantified using SMART tracking software. Fecal boli counts were also collected. The open field was thoroughly cleaned with a 70% EtOH solution between mice.

### Arc immunohistochemistry

A separate cohort of mice underwent fear conditioning, EtOH or control i.p. injections, and were presented with the 3-kHz novel tone stimulus (fear generalization test experiment), using identical procedures to those described above (Control n = 6 and EtOH n = 7). For a comparison of baseline or context-dependent Arc expression, another group (n = 4) was added that underwent fear conditioning and received control injections, but on the test day, explored context B for the identical amount of time as the treatment groups (EtOH and Control groups), but was not presented the novel 3-kHz tone (No tone group). Exactly 90 minutes following the fear generalization test (presentation of the 3-kHz novel tone or no tone control), mice were injected (i.p.) with a ketamine/xylazine cocktail solution (100:10 mg/mL) and transcardially perfused with ice cold 1X PBS, followed by ice cold 4% paraformaldehyde in 1X PBS (7.4 pH). The time point for harvesting the brain following cued fear memory retrieval for Arc immunohistochemistry (IHC) was based on several previous reports^31,32^. Brains were removed and stored in 4% PFA overnight then transferred to 1X PBS and stored at 4 °C until vibratome sectioning (no longer than 4 days).

Brains were sectioned (40 *µ*m) coronally on a vibratome (VT1200, Leica Biosystems Inc., Buffalo Grove, IL USA). Every other section was collected in a well plate (no more than 10 sections/well) in 1X PBS (7.4 pH) for free-floating immunohistochemistry. Immunohistochemical staining was counterbalanced across experimental conditions. Sections were rinsed in 1X PBS, then blocked in a 1X PBS/1% bovine serum albumin (BSA)/0.2% Triton-X solution for 30 min to reduce non-specific binding. Sections were then incubated for 24 hours on an orbital shaker in Arc (C-7) mouse monoclonal antibody (1:100) (Cat# sc-17839, RRID: AB_626696, Santa Cruz Biotechnology, Santa Cruz, CA) at room temperature. The next day, sections were rinsed in 1X PBS before a 1-hour incubation on an orbital shaker in anti-mouse biotinylated IgG (1:200) (Vector Laboratories, Burlingame, CA USA) at room temperature. Sections were rinsed again in 1X PBS then incubated in an ABC kit (Vectastain, Vector Laboratories, Burlingame, CA USA) for 1 hour on an orbital shaker at room temperature. Sections were rinsed again in 1X PBS, and then incubated in DAB peroxidase substrate (Vector Laboratories, Burlingame, CA USA) for exactly 2 min. Sections were then rinsed in 1X PBS and mounted onto gel-coated slides. Sections were dehydrated first in a graded series of EtOH concentrations, and then in xylenes, before cover-slipping with DPX.

### Arc expression analysis

For the quantification of Arc-immunopositive (+) cells, the experimenter was blind to the experimental conditions. All cell counting was conducted using bright-field microscopy (Axio Imager.M2, Zeiss, Thornwood, NY USA) and a 250 × 250 *μ*m counting frame. For each brain region, manual cell counts were conducted bilaterally in six hemispheres per mouse using Neurolucida and NeuroExplorer software (MBF Bioscience, Williston, VT USA). Non-consecutive sections were used for sampling to avoid double-counting. Each brain region was first identified under a 2.5X objective and the counting frame positioned over the region of interest (ROI). Importantly, the counting frame was positioned at a consistent ROI location across sections using anatomical landmarks in conjunction with a mouse brain atlas^44^. The sections chosen for counting were spaced evenly and sampling was not conducted in the rostral- and caudal-most regions of the ROI. All Arc+ cell counting was conducted under a 20X/0.5 NA objective (200X final magnification) with Koehler illumination principles applied. Arc+ cell counting was conducted in the shallow and deep layers of the prelimbic (PL) and infralimbic (IL) cortex. The distinct forceps minor of the corpus callosum was used as an anatomical landmark to locate the PL and IL. The center point of the counting frame was consistently positioned ~150–200 *μ*m (shallow layers) and ~550–600 *μ*m (deep layers) from the lateral surface of the cortex. Arc+ cells in the PL and IL were counted in slices between rostrocaudal levels 2.0 and 1.5 mm (bregma). Amygdala-centric anatomical landmarks, including the rhinal fissure and external capsule, were used to locate the LA, BA, and CeA. Arc+ cells in the LA, BA, and CeA were counted from slices between rostrocaudal levels −1.3 and −2.2 mm (bregma).

### Designer Receptors Exclusively Activated by Designer Drugs (DREADDs)

A separate cohort of mice were stereotaxically microinfused bilaterally with pAAV-CaMKIIa-hM3D(Gq)-mCherry (AAV8; viral particles (vp)/mL titer ≥ 3 × 10^12^) into the IL (pAAV-CaMKIIa-hM3D(Gq)-mCherry was a gift from Bryan Roth (Addgene plasmid # 50476)). Mice in the control group were injected with pAAV-CaMKIIa-EGFP (AAV5; titer ≥ 3 × 10¹² vp/mL). The CaMKII promoter enables selective transgene expression in cortical pyramidal neurons^45^. The volume of the AAV was 100–150 nL/hemisphere. AAV microinjections were conducted 1–2 weeks prior to fear conditioning (>3 weeks prior to Clozapine-N-oxide (CNO) injection). Animals were anesthetized with 4% isoflurane in oxygen (flow at 1.25 L/min), followed by maintenance with 1.25–2.5% inhaled isoflurane throughout the surgery. The anti-inflammatory carprofen (0.1 mg/10 g) was provided during surgery. Coordinates for the IL were AP: +1.70, ML: ±0.35, DV: -2.75^44^. Following microinfusion, the incision was disinfected then closed with Vetbond© tissue adhesive. After surgery, animals were left to recover on a heating pad with oxygen flowing, then placed in a clean home cage and individually housed for 1–2 weeks. Mice were re-group housed at least 5 days prior to behavioral testing. Mice underwent fear conditioning and EtOH exposure using identical methods to those described above. Mice were systemically injected (i.p.) with CNO (5 mg/kg; Hello Bio., Princeton, NJ) suspended in 0.9% saline (1 mg/ml) 45 min prior to the generalization test. CNO is used to selectively activate DREADDs^46^. The time point for CNO administration prior to the CS-retrieval test was based on *in vivo* electrophysiology data showing peak activity 45–50 min following systemic injection^47^. For the generalization test, mice were placed into context B for 180 sec prior to presentation of the 3-kHz novel tones (10 times, 5 sec ITI). The next day, all mice were systemically injected (i.p) with 0.9% sterile saline (control), placed in context B for 180 sec, then presented with the 3-kHz tones (10 times, 5 sec ITI). We hypothesized that any behavioral effects observed following CNO, would diminish in response to the control injection on the day 12 test.

### Efficacy of hM3D(Gq)-DREADD excitation: c-fos fluorescent immunohistochemistry

The efficacy of hM3D(Gq)-DREADD neuronal excitation in the mPFC was tested in a separate cohort of mice (n = 7). Mice were stereotaxically microinjected with pAAV-CaMKIIa-hM3D(Gq)-mCherry or pAAV-CaMKIIa-EGFP in the IL (100–150 nL volume) at least 14 days prior to a systemic injection of CNO (5.0 mg/kg). Peak c-fos induction has been demonstrated ~60–120 min following experience-induced neuronal activity in the rodent brain^48^. Based on evidence showing the peak of CNO activation at 45 min post systemic injection^49^, mice were removed from the home cage, systemically injected (i.p.) with 5.0 mg/kg CNO 135 min prior to anesthetization (ketamine/xylazine solution) and intracardial perfusion with 4% PFA for fluorescent immunohistochemistry. Sections were cut at 40* µ*m for free-floating immunohistochemistry. Sections were first rinsed, then blocked in 1X PBS/1% bovine serum albumin (BSA)/0.2% Triton-X solution for 30 min on an orbital shaker. Next, sections were incubated on an orbital shaker overnight at room temperature in a c-fos rabbit polyclonal antibody (1:500) (Cat# RPCA-c-Fos-AP, RRID: AB_2572236, EnCore Biotechnology, Gainsville, FL USA). Next, sections were rinsed in 1X PBS then incubated in a goat anti-Rabbit IgG (1:250) (Alexa Fluor 488 or 594, ThermoFisher, Waltham, MA USA) at room temperature for 1 hour on an orbital shaker. Sections were then washed and mounted on gel-coated slides with dapi-fluoromount-G (Invitrogen, Carlsbad CA USA) for imaging. Images were captured under a 20x objective using a fluorescent microscope (Nikon E400, Nikon Instruments, Amsterdam, NL). C-fos+ cells were manually counted (Fiji-ImageJ open source) in the IL in a 250 × 250 µm counting frame area with AAV+ expression and an adjacent area of the mPFC with AAV- expression from six hemispheres per mouse (mCherry n = 4; EGFP n = 3). The number of c-fos+ cells in the AAV+ area was divided by the number of c-fos+ cells in the AAV- region and statistically compared (mCherry vs. EGFP) using a KS-test.

### Behavioral quantification

A camera positioned directly above the fear conditioning chambers recorded digital video. The video was analyzed offline using a video tracking system (SMART v3.0, Panlab, Harvard Apparatus, Barcelona, Spain). Immobility (“freezing”) was used as a behavioral measure of a conditioned defensive reflex (“fear”). Freezing was operationally defined as immobility, except for respiration. Freezing behavior was scored only when bouts of immobility lasted >1 sec. For each test, freezing duration during habituation, CSs, and ITIs were averaged and converted into percentages of total time. To verify the accuracy of the tracking software for quantifying freezing behavior, a subset of mice was scored for freezing by a trained observer that was blind to the experimental conditions and compared with the results of the automated tracking software. Results revealed a remarkably high inter-rater reliability (SMART vs. trained human rater; R^2^ = 0.99). A discrimination index was used to quantify the degree of cued fear memory discrimination and generalization^42^. The discrimination index was generated by dividing the mean of the target (T) stimulus (5-kHz) by the sum of T and the non-target (N) stimulus (3-kHz) [T/(T + N)]. In the discrimination index, a value of 1 indicates discrimination and a value of 0.5 indicates generalization. In addition to the standard conditioned freezing measure, we conducted a multi-measure analysis of behaviors (alternate to freezing) present during the Pre-CS and CS intervals using both manual and automated video tracking approaches^50^. The multi-measure analysis was applied to the generalization data only. We included path length (locomotor activity) and freezing bouts during CS presentations using SMART software. For freezing bouts, seconds spent freezing were divided by the number of bouts to generate a mean freezing/bout duration. Manually scored measures included the rearing, grooming, and circling (right or left) bouts. Tail-rattling was also analyzed; however, this behavior was under-represented (4 instances across all CSs and all mice) and removed from the analysis.

### Statistical Analyses

#### Behavior

For all analyses, the independent variable was treatment (EtOH vs. Control). The dependent variable was freezing % (mean freezing percentage for the pre-CS period, CS presentations or ITIs). The freezing % was averaged (binned) across 10 CS presentations. Prior to all analyses, mean values outside a step of 1.5 x the interquartile range were designated outliers and removed from analysis. Homogeneity of variance was checked using Levene’s test. Mixed repeated-measures ANOVA (RMANOVA) was used to analyze how freezing % changed over time (bins; within-group factor) and treatment (between-group factor) groups. Follow-up comparisons were conducted using one-way ANOVA. If the distribution violated the assumption of normality, the Welsh F-ratio was used. Data for the discrimination index was analyzed using two-way ANOVA (Treatment × kHz) followed by Bonferroni corrected t-tests. Multi-measures of behavior during fear generalization testing (e.g., circling, rearing, grooming) were analyzed using Fisher’s exact test.

#### Pattern analyses of prefrontal-amygdala Arc expression

Multivariate analysis of variance (MANOVA) was used to test the statistical relationship among *Arc* expression levels in PL (deep and shallow), IL (deep and shallow), LA, BA, and CeA (7 factors) and treatment groups (No tone, EtOH, and control groups). A significant value for the conservative Pillai’s Trace test statistic was followed up by ANOVAs. Assumptions of multivariate normality and equality of covariance matrices were checked using Levene’s test and Box’s test, respectively. Violations of Levene’s test were followed up with a Welsch test. All Arc expression data were log-transformed prior to analysis. Next, functional discriminant analyses (DA) was applied to evaluate how the pattern of Arc expression across the mPFC-BLA brain regions discriminates the groups. DA is a classification method that extracts the optimal combination of variables (a dimension) that discriminates the groups. For DA, the grouping variable were the conditions (No tone, EtOH, Control) and the independent variables (discriminate variates) were the Arc expression values across mPFC-BLA regions (IL, PL, LA, BA, CeA). The result of DA is a set of standardized canonical discriminant function coefficients that provide the relative contribution of Arc expression in each brain region to the conditions (No tone, Control, EtOH). Variables with correlations greater or less than 0.3 were reported and interpreted.

#### CaMKII-hM3Dq-DREADD IL excitation

If CaMKII-EGFP or CaMKII-hM3Dq expression was not detected in the IL, mice were excluded from analysis. RMANOVA was used to analyze freezing behavior in the DREADDs study. Follow-up analyses were based on a priori predictions using an orthogonal planned comparisons approach (three comparisons). First, we hypothesized that EtOH would enhance cued fear memory generalization, replicating previous findings (EGFP-EtOH vs. EGFP-no EtOH). Next, we hypothesized that excitation of the IL reverses EtOH-induced overgeneralization (hM3Dq-EtOH vs. EGFP-EtOH). Finally, we hypothesized that IL excitation improves discrimination (hM3Dq-no EtOH vs. EGFP-no EtOH). This hypothesis was based on previous data implicating the IL in fear memory discrimination and generalization^42^.

For all analyses, statistical significance was set at *p* ≤ 0.05 (two-tailed). Statistical analyses were carried out using SPSS Software (v. 22, IBM, Armonk, NY). Data are represented as the mean ± the standard error of the mean (SEM). Group sizes can be found in the text (Methods and Results section) and duplicated in the figure captions.

## Results

### Cued fear memory generalization

**Figure 1:**
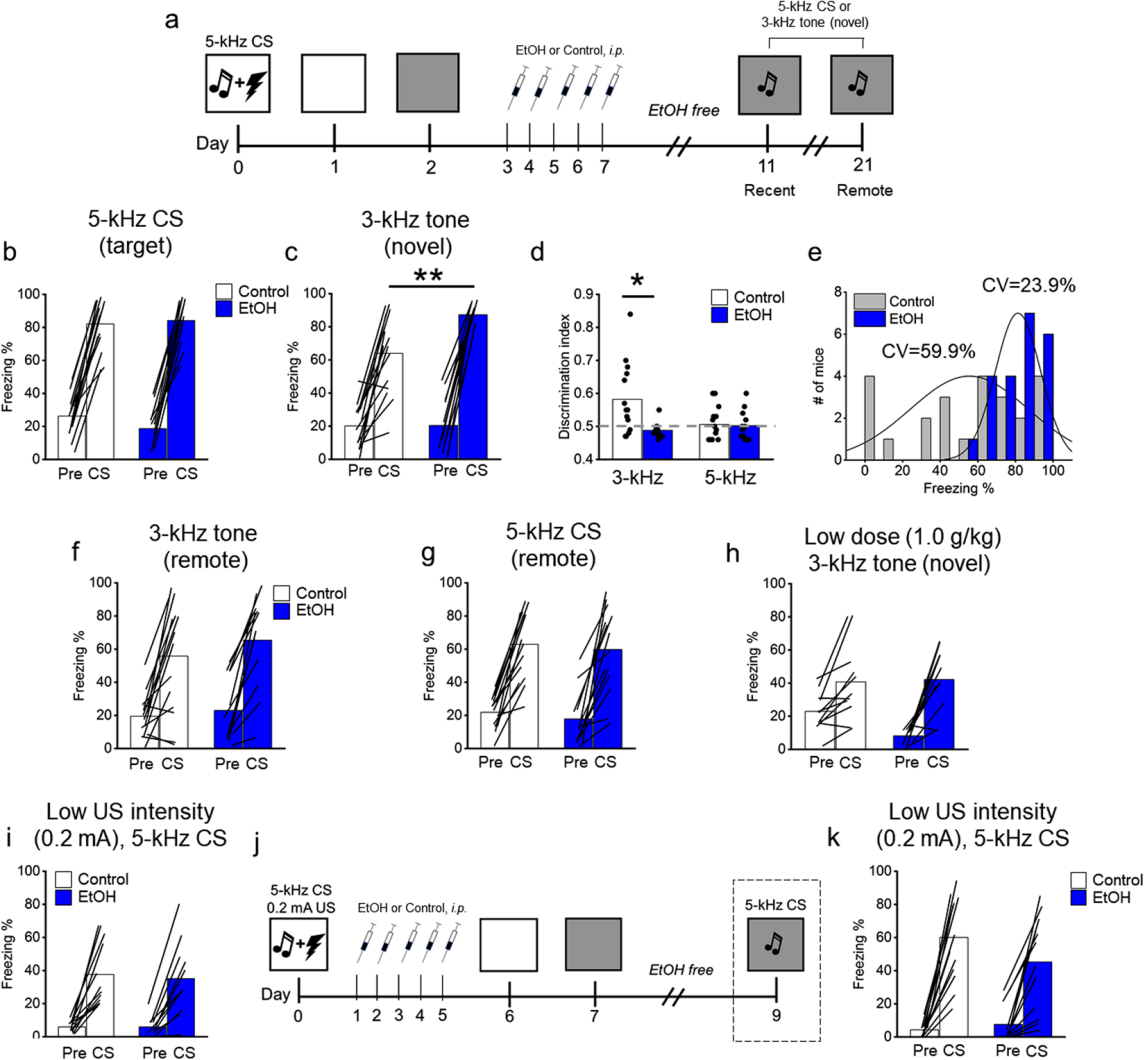
Chronic EtOH following fear conditioning increases cued fear memory generalization (**a**) Schematic depicting the experimental design for the generalization study (**b**) EtOH did not impact the retrieval of the CS (5-kHz). (**c**) EtOH following fear conditioning increased conditioned freezing to a novel tone (3-kHz). (**d**) Mice discriminated the 3-kHz novel tone stimulus from the 5-kHz CS. EtOH eliminated stimulus discrimination. The dashed line indicates complete generalization. (**e**) The coefficient of variation (CV) was greater in the control vs. EtOH groups. (**f**,**g**) There were no effects of EtOH at remote time points (14 days) following EtOH exposure. (**h**) A lower dose of EtOH (1.0 g/kg) did not impact retrieval of the 3-kHz novel tone stimulus. (**i**) The retrieval of a cued fear memory formed using a weaker US intensity (0.2 mA) was also not impacted by EtOH. (**j**) Schematic depicting the experimental design for the immediate EtOH study. (**k**) EtOH timed 24 hrs following learning did not impact fear memory retrieval. n = 10–14/group, **p < 0.01, *p < 0.05, one-way ANOVA.

Cued fear memory expression in response to the original “target” CS (5-kHz) or a novel tone (3-kHz) stimulus (generalization) was tested in separate cohorts of mice (n = 12–15/group). Mixed RMANOVA revealed a significant Treatment X kHz X Time interaction for the novel tone (*F*[4, 208] = 2.5; *p* = 0.04), but not CS (Fig. S1). Subsequent ANOVAs (Bonferroni corrected) run on each time bin revealed treatment effects at CS 1–10 only. Because treatment effects were only uncovered during CS 1–10, subsequent analyses were confined to CS 1–10 mean values. There was no effect of EtOH on the retrieval of the target 5-kHz CS (Fig. 1b). However, mice in the EtOH group exhibited greater freezing in response to a novel 3-kHz tone relative to controls (*F*[1, 15.8] = 11.8; *p* = 0.003) (Fig. 1c), indicating increased cued fear memory generalization after chronic EtOH exposure.

Next, a discrimination index was calculated and analyzed to determine the degree of EtOH-induced cued fear memory discrimination and generalization. A two-way ANOVA on the discrimination index revealed a significant kHz X Treatment interaction (*F*[1, 49] = 6.01; *p* = 0.018). Subsequent analysis of the control group revealed a greater discrimination index in the 3-kHz group vs. 5-kHz (t-test; *p* = 0.02), confirming the 3-kHz novel tone stimulus was perceptually discriminated from the 5-kHz CS. In the EtOH group, the discrimination index for the 3-kHz and 5-kHz frequencies was statistically equivalent (t-test; *p* = 0.32), indicating that EtOH increased cued fear memory generalization (Fig. 1d). There were no group differences at remote time points or pre-CS periods for either frequency (Fig. 1f,g). The effect of EtOH on cued fear memory generalization was replicated several times in subsequent immunohistochemistry and chemogenetic experiments (see below). This allowed for an analysis of the dispersion for each group in a larger data set. Results revealed a wide frequency distribution in controls, as indicated by the coefficient of variance (CV = 59.9), compared with the EtOH group (CV = 23.9) (Fig. 1e).

To ascertain whether alternate behaviors, outside of freezing, may have differentiated the EtOH and control treatment groups during the presentation of the novel tone (3-kHz), we conducted an additional “multi-measure” analysis of behavior in the same cohort of mice. Fisher’s exact test revealed no significant differences among rearing, grooming, circling, or bias in the direction of circling, behaviors (Fig. S2). In addition, the number of freezing bouts and freezing duration per bout during the CS was analyzed. There were no differences in the number of freezing bouts between treatment groups. However, when the CS-elicited freezing/bout was calculated, mice in the EtOH group froze significantly longer per bout relative to controls (*F*[1, 22.3] = 6.5; *p* = 0.018). These results indicate that EtOH did not increase the number of freezing instances per se, but instead, increased the duration spent freezing per freezing bout (Fig. S2).

To determine whether a lower, but pharmacologically relevant, EtOH dose impacts cued fear memory generalization, a separate cohort of mice (n = 13–15/group) was fear conditioned and tested for generalization using identical procedures to those outlined above but administered a lower (1.0 g/kg) EtOH dose. The dose was chosen based on previous data showing an acute 1.0 g/kg EtOH dose disrupts fear conditioning^51^. Results revealed no effect of low dose EtOH on fear memory generalization (Fig. 1h). Low dose EtOH however, did produce a subtle degree of weight loss, although not to the extent of the 2.5 g/kg dose (Fig. S3).

EtOH, and particularly EtOH withdrawal, is known to induce lasting non-specific locomotor and/or anxiety-related behaviors^52^, which may have influenced the cued fear memory generalization results. To address this question, we EtOH-exposed a separate cohort of mice (n = 5–7/group) and tested them on the novel open field at the identical time point post-EtOH as in all previous experiments. Results revealed no treatment effects in open field exploration or duration in the center (Fig. S4). To provide another measure of anxiety-like behavior, we ran a subset of mice (n = 11–13/group) on the elevated plus maze approximately 4 hrs following a cued fear generalization test (see above). Results revealed no effects on open/closed arm time or entries (Fig. S5). Further, there were no effects of treatment on distance traveled or immobility. Together, results from the novel open field and elevated plus maze indicate EtOH did not produce non-specific effects on locomotor activity or anxiety-related behavior.

### Cued fear memory expression

In first set of experiments, no effect of EtOH was found on the expression of the original “target” 5-kHz CS. To further study the question of whether EtOH effects the expression of the original cued fear memory, we conducted a series of follow-up studies. In the previous experiments, the intensity of the US was relatively high (0.6 mA), producing relatively high freezing levels (~80%), which may have occluded the effects of EtOH on cued fear memory expression (i.e., a putative “ceiling effect”). To address the possibility that a reduction in the amplitude of the US may un-mask an effect of EtOH on fear memory expression, we replicated the cued fear memory expression experiment, but with a lower US amplitude (0.2 mA) during training in a separate cohort of mice (n = 10–11/group). We first verified that a 0.2 mA US produces reduced CRs during presentation of the 5-kHz CS (36.6 ± 4.3%) than 0.6 mA (82.1 ± 3.9) using the same number of pairings (three). Results revealed no effect of EtOH on a memory formed using a weaker US intensity (Fig. 1i). These results provide evidence that the expression of cued fear memories, formed using relatively high (0.6 mA) and low (0.2 mA) US intensity, are not impacted by EtOH exposure following training.

In the second experiment, we considered the timing between EtOH exposure and cued fear memory retrieval to the “target” 5-kHz CS. There were four EtOH-free days between the cessation of EtOH exposure and cued fear retrieval (Fig. 1a), a time window which may have diminished the lasting effects of chronic EtOH exposure. To test this possibility, a separate cohort of mice (n = 14/group) was tested using a shortened (24 hr) interval between training and EtOH exposure (Fig. 1j). To eliminate potential ceiling effects, a training protocol of lower memory intensity (0.2 mA US) was used (see above). Results did not uncover a significant effect of EtOH on pre-CS or CS freezing, although a statistical trend (ANOVA, *F*[1, 26] = 3.2; *p* = 0.08) towards a reduction in CS freezing in the EtOH group was detected (Fig. 1k).

### Arc/arg3.1 immunohistochemistry

**Figure 2:**
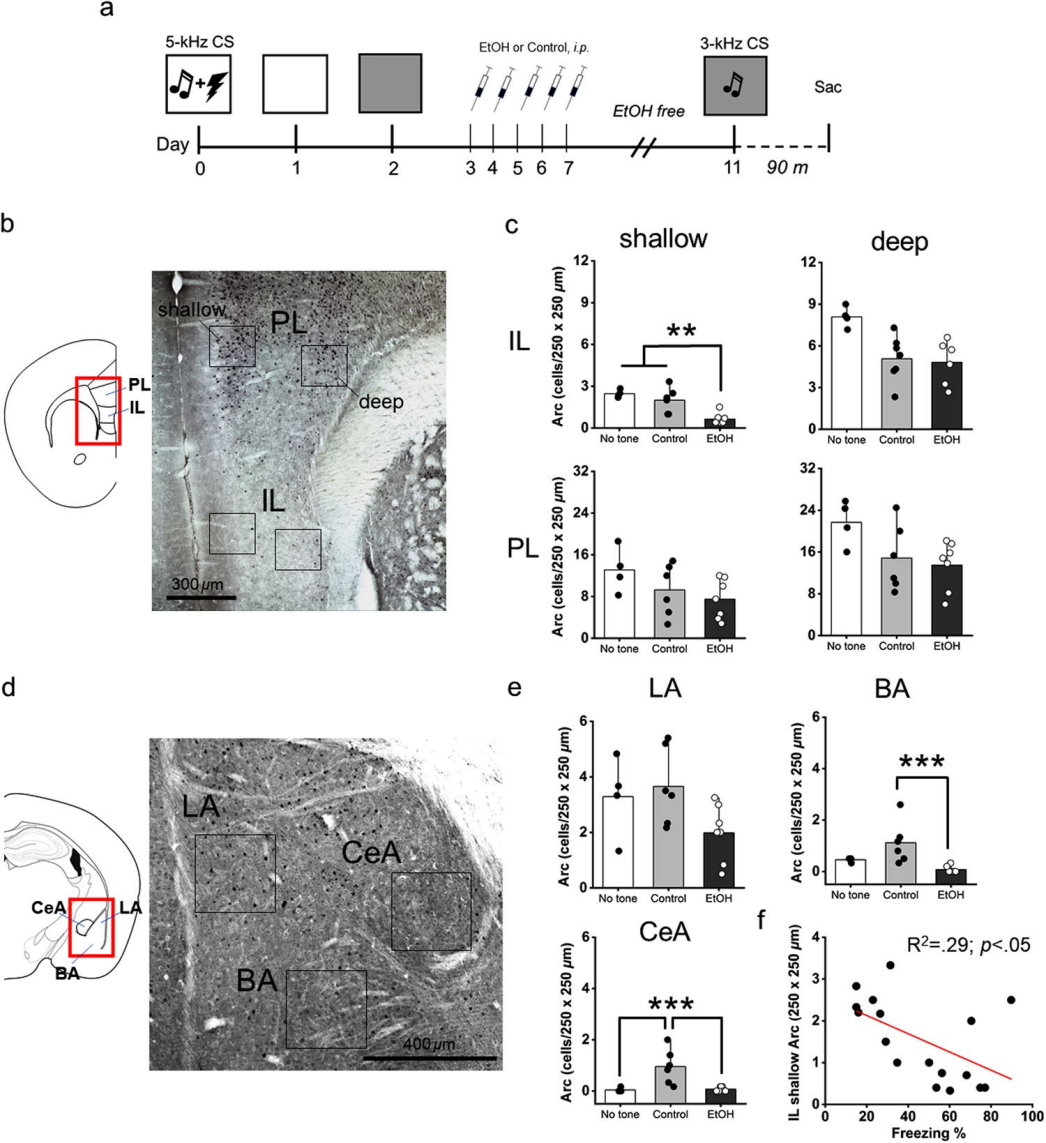
Arc expression following retrieval of a novel tone frequency in the mPFC and BLA. (**a**) Schematic depicting the experimental design for the Arc immunohistochemistry study. (**b**) Left: Atlas image depicting the location of the mPFC. Right: Representative photomicrograph depicting Arc immunohistochemical staining and relative location of the counting frames (250 × 250 *µ*m) in the shallow and deep layers of the IL and PL cortex. (**c**) There was a significant reduction in Arc expression in the EtOH group vs. the No tone and Control conditions in the IL shallow layers only. (**d**) Left: Atlas image depicting the location of the amygdala. Mouse brain atlas images modified from^44^. Right: Representative photomicrograph depicting Arc immunohistochemical staining and relative location of the counting frames (250 × 250 *µ*m) in the LA, BA, and CeA. (**e**) In the BA, there was a significant reduction in Arc expression in the EtOH vs. Control group. In the CeA, Arc expression in the Control group was greater than the No tone and EtOH groups. (**f**) A negative relationship between IL shallow layer Arc expression and conditioned freezing performance was detected (R^2^ = 0.29, *p* < 0.05, regression line in red). No other correlations between freezing behavior and Arc expression were uncovered across mPFC or BLA. n = 4–7/group, **p < 0.01, ***p < 0.001. IL, infralimbic; PL, prelimbic; LA, lateral amygdala; BA, basal amygdala; CeA, Central nucleus of the amygdala.

Stimulus generalization is linked with mPFC and BLA functionality and plasticity^42,53–58^. Further, chronic EtOH has reliably been shown to produce neuroadaptations in both the mPFC and BLA^17,59–65^. To determine loci of EtOH-driven neuroadaptations in a mPFC-BLA circuit that are associated with the overgeneralization of fear memory (Fig. 2a), we measured the expression of the activity-regulated cytoskeletal protein Arc/arg3.1 in the mPFC (PL and IL subregions) (Fig. 2b) and BLA (LA, BA, CeA subnuclei) (Fig. 2d) using immunohistochemistry.

MANOVA revealed a significant effect of treatment (n = 4–7/group) on Arc expression in a cortico-amygdala circuit *(V* = 1.6, *F*[14, 18] = 5.8; *p* < 0.001). Follow-up ANOVAs revealed significant effects of treatment on Arc expression in the IL shallow layer (*F*[2, 14] = 16.9; *p* < 0.001) (Fig. 2c), BA (*F*[2, 14] = 20.4; *p* < 0.001) and CeA (*F*[2, 14] = 17.1; *p* < 0.001) only (Fig. 2e). In the IL shallow layers, post hoc analysis (Scheffe) showed less Arc expression in the EtOH vs. No tone (*p* = 0.001) and EtOH vs. Control (*p* = 0.002) groups. In the BA, post hoc analysis revealed less Arc expression in the EtOH vs. Control (*p* < 0.001) groups. Finally, in the CeA, post hoc analysis uncovered less Arc expression in the EtOH vs. Control (*p* < 0.001) and No Tone vs. Control group (*p* < 0.001).

MANOVA was followed-up with discriminant analysis (DA) to elucidate the nature of the relationship among mPFC - BLA Arc expression and how the relationship contributes to treatment differences. DA revealed a prominent underlying dimension in the data set that segregated the EtOH vs. Control treatment groups. The relative contribution of the BA (loading value = 78), then shallow IL (0.65), followed by the CeA (0.48) influenced differences in Arc expression that depended on treatment (EtOH vs. Control groups). These findings implicate a BA > IL > CeA pattern of Arc expression distinguished the EtOH-exposed group from the Control groups.

Notably, a second dimension was extracted that segregated the “No Tone” from the 3-kHz groups, indicating this dimension was related to associative learning. In this case, the shallow IL (loading value = −0.52), followed by the deep IL (−0.38), and CeA (0.31) segregated the groups. The positive and negative signs suggest opposing influence of IL and CeA Arc expression differentiated conditioned fear responses to novel tone stimuli from the “No tone” control. Overall, this finding supports a role for an IL – CeA network in fear memory discrimination and generalization^42,66,67^.

Finally, relationships between Arc expression in the mPFC – BLA and freezing behavior were conducted. Correlations revealed a significant negative relationship between Arc expression in the IL shallow layer and conditioned freezing (R^2^ = 0.29, *p* < 0.05). No other relationships were detected (Fig. 2f).

### Designer Receptors Exclusively Activated by Designer Drugs (DREADDs)

**Figure 3:**
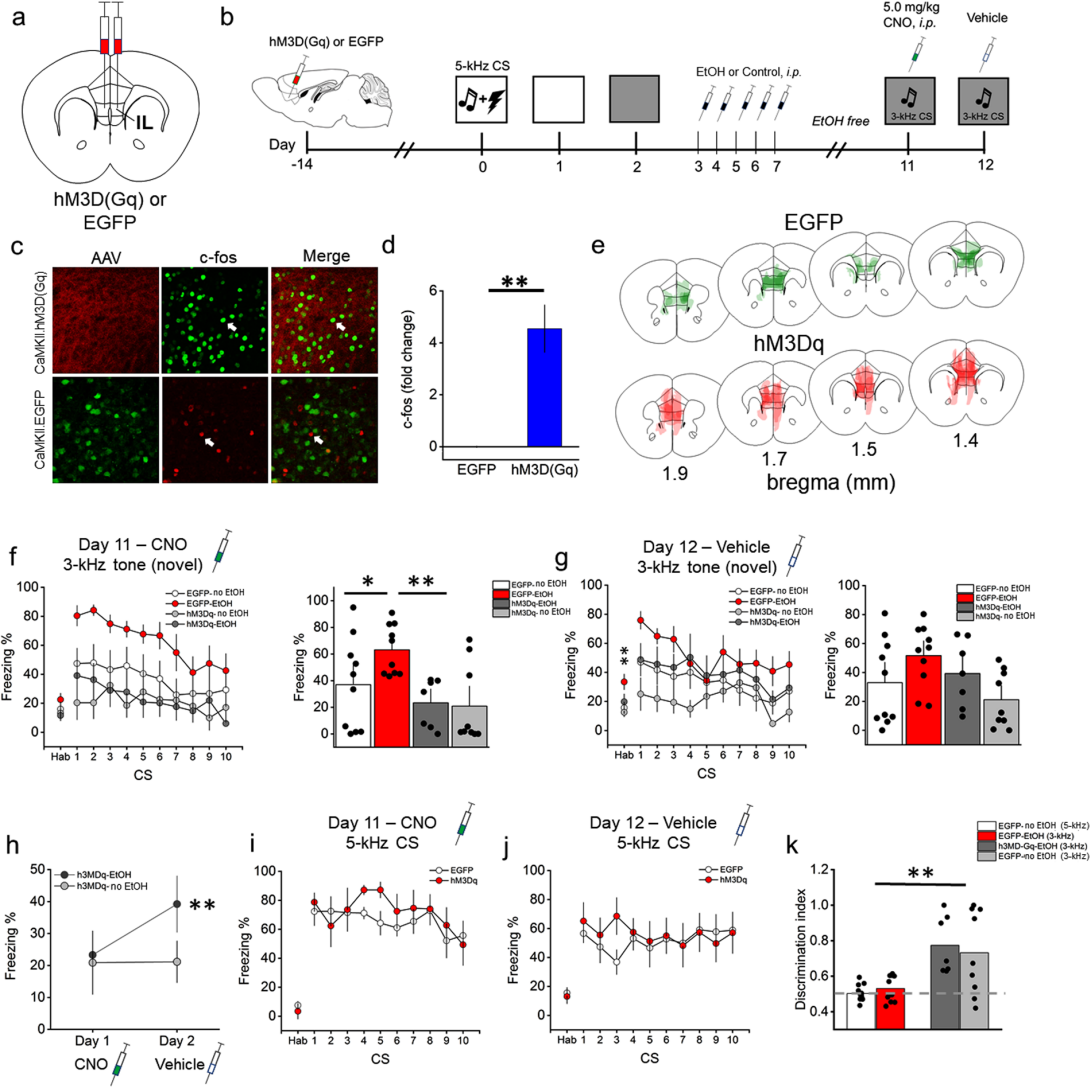
Chemogenetic stimulation of the IL reverses EtOH-induced overgeneralization. (**a**) Diagram depicting the location of the pAAV-CaMKIIa-hM3D(Gq)-mCherry (hM3Dq) or pAAV-CaMKIIa-EGFP (EGFP) in the IL. (**b**) Schematic depicting the experimental design for the DREADDs fear memory generalization study. (**c**) Representative confocal images of AAV expression (left panel), c-fos FIH (middle panel) and AAV/c-fos colocalization (63X/1.20 N.A. water immersion objective). Arrows indicate c-fos-positive cell. (**d**) There was 4-fold increase in c-fos expression in the hM3Dq (n = 4) vs. EGFP (n = 3) groups, *p* = 0.008. (**e**) The extent of AAV expression in the mPFC across four coronal planes is depicted. The pattern of AAV expression was digitally reconstructed, shaded (10% opacity), and aligned in a common stereotaxic group space across all mice. Relatively darker shaded regions show greater overlap across individuals. Mouse brain atlas images modified from^44^. (**f**) Conditioned freezing response to the novel tone (3-kHz) increased after EtOH administration. IL stimulation reduced freezing levels in EtOH-exposed mice. (**g**) Freezing was equivalent across groups after vehicle injection. (**h**) Freezing was stable across CNO (day 11 test) and vehicle injections (day 12 test) in the h3MDq-no EtOH group. Freezing increased across CNO (day 11 test) and vehicle injections (day 12 test) in the h3MDq-EtOH group. (**i**,**j**) There were no differences in freezing in response to the original CS across CNO (day 11 test) and vehicle injections (day 12 test). (**k**) The discrimination index approached 0.5 and was equivalent in the 5-kHz and EGFP-EtOH, indicating generalization. The discrimination index was statistically equivalent in the h3MDq-EtOH and EGFP-no EtOH groups, but greater than the EGFP-EtOH and 5-kHz groups, indicating discrimination. The dashed line indicates complete generalization. n = 7–11/group. *p < 0.05, **p < 0.01.

EtOH targets the prefrontal cortex, driving neuroadaptations associated with behavioral/cognitive changes, including loss of flexibility and inhibitory control. To establish a causal role for EtOH-induced neuroadaptation in the IL and fear memory performance, we used a chemogenetic approach (Designer Receptors Exclusively Activated by Designer Drugs; DREADDs). The present Arc IHC results revealed a reduction in Arc expression in the IL after EtOH exposure that negatively correlated with fear memory expression (Fig. 2c,f). Considering that IL activity has been linked with CR suppression^68–70^, and EtOH-induced reduction in IL NMDAR-mediated current was associated with impaired fear extinction retrieval^17^, we hypothesized that stimulation of the IL would reduce EtOH-induced overgeneralization in response to the novel tone. The efficacy of our excitatory DREADDs system was first tested using c-fos following CNO in hM3Dq and EGFP mice. We found a 4-fold increase in c-fos expression in hM3Dq mice relative to the controls, confirming the efficacy of the excitatory DREADDs system in the mPFC (Fig. 3d).

On the day 11 test (CNO injection), RMANOVA showed a significant main effect of treatment (*F*[3, 32] = 4.8; *p* = 0.007), but no interaction with Time (n = 7–10/group) (Fig. 3f). Planned comparisons revealed a significant increase in freezing in the EGFP-EtOH relative to the EGFP-no EtOH group (*p* = 0.04), replicating the previous finding of EtOH-induced cued fear memory overgeneralization. There was a significant decrease in freezing in the hM3Dq-EtOH group relative to the EGFP-EtOH group (*p* = 0.005), indicating Gq-DREADD-mediated excitation of the IL pyramidal cells reversed EtOH-induced fear memory overgeneralization. Finally, no differences were detected between the EGFP-no EtOH and hM3Dq-no EtOH groups, indicating IL excitation did not reduce freezing beyond control levels. There were also no differences in pre-CS freezing for any group.

Consistent with the hypothesis that behavioral effects would be expected to reverse in response to control injection on the day 12 test (control injection), RMANOVA revealed no effects of treatment on 3-kHz tone-elicited freezing levels (Fig. 3g). However, there was a significant treatment effect on pre-CS freezing (*F*[3, 32] = 4.5; *p* = 0.01). Mice in the EGFP-EtOH group exhibited increased pre-CS freezing relative to the EGFP-no EtOH group (Scheffe; *p* = 0.05) and hM3Dq-no EtOH groups (Scheffe*; p* = 0.02). To verify the specificity of the DREAADs manipulation, the day 11 test (CNO) versus day 12 test (vehicle) data were compared. Results revealed a significant increase in freezing in the hM3Dq-EtOH group from day 11 test to day 12 test (paired t-test; *p* = 0.01), but not in the EGFP-no EtOH group (Fig. 3h), verifying the specificity of the synthetic manipulation of the IL.

In the next experiment, we addressed the possibility that IL stimulation suppresses CS-elicited freezing, independent of generalization. To test this question, a separate cohort of mice (n = 6–7/group) underwent procedures identical to those described above, except were exposed to the control regimen only following training and received the target 5-kHz CS during the retrieval test. We did not include an EtOH exposure group because we were interested in the direct effects of IL stimulation on the recall of the target 5-kHz CS, independent of EtOH exposure. Results revealed no difference in freezing between hM3Dq and EGFP groups (Fig. S6) on the day 11 test (CNO) (Fig. 3i) or day 12 test (control) (Fig. 3j), showing that IL stimulation does not reduce freezing levels after presentation of the “target” 5-kHz CS. These data suggest that IL stimulation selectively improves fear memory discrimination after EtOH exposure. Next, a discrimination index was calculated and analyzed to determine the overall degree of discrimination and generalization in the DREADDs experiment (Fig. 3k). Two-way ANOVA on the discrimination index revealed a significant kHz X Treatment interaction (*F*[3, 36] = 8.23; *p* < 0.001). Post hoc analysis (Scheffe) revealed the discrimination index for the EGFP-no EtOH (5-kHz) group (0.50 ± 0.01) was statistically equivalent to the EGFP-EtOH (3-kHz) (0.53 ± 0.02) (*p* = 0.92), indicating robust generalization of the 3-kHz tone following EtOH. The discrimination indices for the hM3Dq-EtOH (3-kHz) (0.77 ± 0.06) and EGFP-no EtOH (3-kHz) groups (0.72 ± 0.07) were also statistically equivalent (*p* = 0.9), but greater than both the EGFP-no EtOH (5-kHz) and EGFP-EtOH (3-kHz) groups (*p* = 0.01), indicating IL stimulation significantly improved cued fear memory discrimination following EtOH exposure (Fig. 3k).

### Cued fear memory extinction

**Figure 4:**
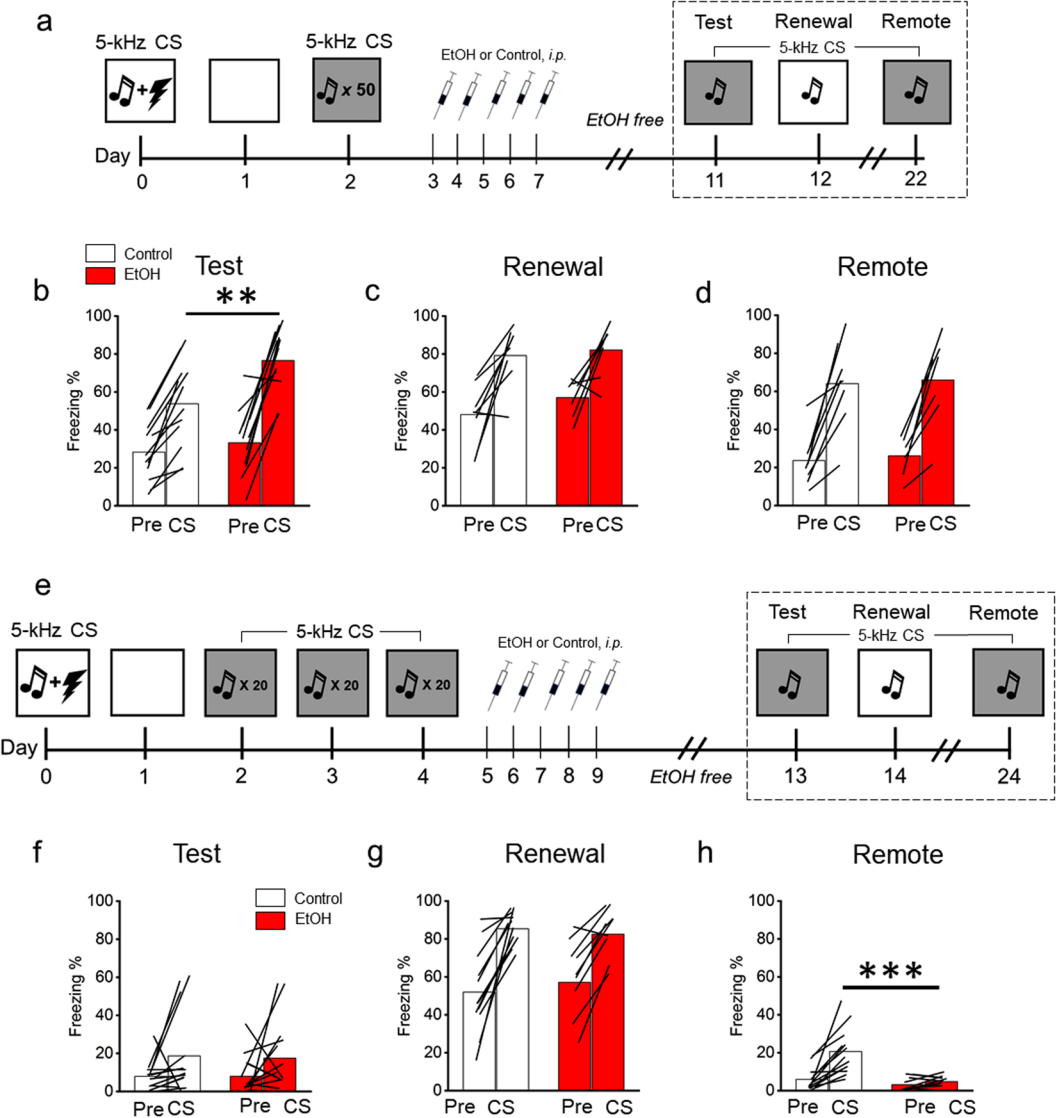
Chronic EtOH following fear extinction impairs retrieval (**a**) Schematic depicting the experimental design for the extinction study (**b**) EtOH following fear extinction increased conditioned freezing. (**c**,**d**) There was no effect of EtOH on context renewal or remote retrieval (15 days following EtOH exposure). (**e**) Schematic depicting the experimental design for the strong extinction study. (**f**–**g**) There was no effect of EtOH on retrieval and context renewal. (**h**) EtOH following strong fear extinction reduced remote conditioned freezing (15 days following EtOH exposure). EtOH n = 14/group. **p < 0.01, ***p < 0.001.

Arc expression measurements following EtOH exposure and cued fear memory retrieval have so far indicated EtOH-induced neuroadaptations targeting the IL and BLA. Further, we found that excitation of the IL reversed EtOH-driven fear memory overgeneralization. Several studies have definitively linked IL – BLA circuitry with fear extinction^69,71^. Given our findings that EtOH impacts the IL, we hypothesized that EtOH might also impair fear extinction retrieval, resulting in more freezing. To test this question, mice underwent fear conditioning and fear extinction. Mice were then administered EtOH or control (n = 13–14/group). After four days without EtOH, the cued fear extinction memory was tested (Fig. 4a). Results revealed increased freezing in the EtOH group compared with controls (ANOVA; *F*[1, 24] = 8.1; *p* = 0.009), providing support for our hypothesis that EtOH impairs fear extinction retrieval, resulting in more freezing (Fig. 4b). Considering the dependence of fear extinction on the IL and BLA, these data further support the hypothesis that EtOH produces neuroadaptations in IL – BLA circuitry. There were no differences in freezing prior to the CS, during the contextual fear memory renewal test, or when tested for CS-retrieval at a remote time frame (Fig. 4b–d). Together, these results indicate that chronic EtOH administered following fear extinction learning selectively augments CS-elicited freezing behavior.

Extinction learning only resulted in ~50% freezing levels during the extinction test. This suggested a relatively “weaker” extinction memory was formed, leaving open the question of how EtOH impacts a relatively “stronger” extinction memory. To test this question, mice (n = 14/group) were presented with 20 CSs in the absence of the US on three consecutive days (“strong” extinction) (Fig. 4e). Mice were then exposed to EtOH or control and the extinction memory was tested the next day. Results revealed significantly lower levels of freezing on the test day after “strong” extinction (19 ± 6% freezing) compared to “weak” extinction (58 ± 5% freezing) (Fig. S7). This indicates the “strong” extinction protocol sufficiently suppressed cued conditioned freezing. Results revealed no differences in conditioned freezing on the test day or during contextual renewal between treatment groups (Fig. 4f,g). However, at the remote time point, there was a significant reduction in freezing behavior in the EtOH group (ANOVA; *F*[1, 22] = 17.8; *p* < 0.001) relative to controls (Fig. 4h). Together, these data suggest that the effects of EtOH on the expression of cued fear extinction are complex and may depend on extinction memory strength.

## Discussion

In most previous work examining how chronic EtOH modifies fear memory expression, EtOH exposure preceded fear conditioning. Here we sought to isolate the effect of EtOH on the expression of fear memories formed prior to EtOH exposure. We report that chronic EtOH exposure following cued fear conditioning selectively impacted retrieval performance. Specifically, EtOH enhanced fear memory generalization while leaving the expression of the original fear memory trace intact. The effects of EtOH on cued fear generalization could not be attributed to augmented context generalization and the effects reversed with the passage of time. EtOH-induced fear memory overgeneralization was associated with a reduction in Arc/arg3.1 expression in the shallow layers of the IL, BA, and CeA. This finding prompted a study using chemogenetic tools to identify the relative functional contribution of EtOH driven-changes in the IL to fear memory overgeneralization. Gq-DREADD-mediated excitation of IL pyramidal neurons reversed EtOH-driven over-generalization, supporting a role for IL output in fear memory precision^42,72^. Together, these findings led to a model in which EtOH-induced neuroadaptations (i.e., putative hypoactivity) in the IL contribute to fear memory overgeneralization. To test this model, we used fear extinction, a task that depends on the integrity of IL functionality^73^. Results showed that in mice exposed to EtOH following fear extinction, extinction retrieval was impaired, supporting the hypothesis that chronic EtOH produces neuroadaptations in IL circuits, leading to exaggerated conditioned fear responses. Considering that extinction impairments^74^, overgeneralized fear^28^, and functional changes in ventral medial prefrontal and amygdala circuits^75^ are thought to contribute to PTSD symptomatology, these data provide new mechanistic insight into how excessive alcohol consumption, following exposure to a traumatic event, can worsen trauma-related symptoms. This idea may, in part, explain poorer or protracted treatment outcomes in those with PTSD^1^.

### EtOH and fear generalization

Generalization refers to conditioned responding to stimuli that may only partially resemble the stimuli that were present during encoding^76^. Here we showed that EtOH administered following fear learning increased cued fear memory generalization. These results support previous findings for EtOH-induced increases in generalization of a learned auditory fear stimulus discrimination^25^ and reduced fast fear discrimination following EtOH exposure during adolescence^21^. One proposed mechanism for stimulus generalization is that it represents a degradative process, akin to forgetting^77^. Based on our findings, EtOH-induced degradation of the original cued fear memory trace is an unlikely interpretation because conditioned responding to the original CS remained intact, even over time. Rather, EtOH-induced increases in cued fear memory generalization may reflect a broadening of plasticity that is normally confined to an encoded stimulus attribute of the CS (i.e., tone frequency)^78,79^. This interpretation is bolstered by the present results showing cued, not context, specific effects of EtOH on fear memory generalization.

The neurocircuitry mediating fear memory generalization is relatively uncharacterized^54,80^. There is evidence the IL plays a general inhibitory role in fear expression^81^ and in learned cued fear discrimination^72^. A recent study showed fear memory generalization was associated with a reduction in Arc expression in the IL and increased Arc expression in the lateral amygdala (LA)^42^. Various other components of the amygdala complex have also been implicated in fear memory generalization and discrimination, including the BA and CeA^57,67,82–87^. There is also evidence that chronic EtOH produces neuroadaptions in an IL – BA – CeA circuit which has been linked with fear memory expression. Specifically, NMDAR-mediated signaling in the IL following EtOH exposure was reduced during late extinction and extinction retrieval^17^. Further, EtOH-driven neuronal activity (c-fos) in the BA, CeA and PL, but not IL, was increased following cued fear memory retrieval^22^. The present data provide a link between EtOH-driven neuroadaptation in the IL and fear memory generalization, and support evidence for overlapping brain systems mediating fear and reward^88,89^.

Chemogenetic excitation of IL pyramidal neurons during retrieval of the novel tone was sufficient to reduce generalization after EtOH exposure. Importantly, IL excitation failed to drive down freezing levels in the control groups and in response to the “target” 5-kHz CS (Fig. 3i), indicating IL activity underlies cued fear memory stimulus generalization and discrimination, and not conditioned freezing per se. In the absence of CNO (Day 12 test), CS-specific freezing effects were reduced. However, pre-CS freezing levels differed (contextual fear), with mice in the EGFP-EtOH group exhibiting more freezing. This finding suggests a new, higher-order conditioned fear memory about the context (S2) was formed, but only after EtOH exposure^90^. Higher-order conditioning was eliminated in the hM3Dq-EtOH group, suggesting that synthetic activation of the IL improved CS (S1) discrimination during training, resulting in a reduction in higher-order (S2) conditioning during testing. Although the IL was targeted, AAV infection was observed, in several cases, outside the IL (Fig. 3e). Therefore, the contribution of additional sites, such as the PL, to the fear memory generalization cannot be completely discounted. Activation of downstream limbic targets of the IL, outside of the BLA complex, may also have contributed to the observed effects, including the thalamus, hypothalamus, bed nucleus of the stria terminalis, hippocampus, and various striatal subregions^91^. Overall, these data identify the IL as a target of EtOH-induced neuroadaptations. Further, these data implicate the IL in cued fear memory generalization and discrimination. An important future experiment will be to determine how driving IL excitation during fear memory retrieval changes the pattern of network plasticity across the IL – BA – CeA circuit^92^.

### EtOH and fear extinction

Fear extinction is thought to represent new learning that creates a parallel inhibitory memory competing for control over the expression of the original fear memory trace^93^. The fact that extinction may represent a new memory, rather than memory erasure (although see^94^), suggests that circuits and synapses recruited and modified during extinction formation and retrieval could be uniquely targeted by pharmacologic intervention^73,95^. Studies examining the impact of chronic EtOH on fear extinction have generally indicated extinction impairment^11,17,18,20,23,24^. However, in all previous studies, EtOH was administered prior to extinction learning, which makes it difficult to parse the specific impact of EtOH on extinction learning, storage, and retrieval^96^. In the present study, EtOH was administered following extinction learning, and the memory subsequently tested, allowing for a more precise interpretation of EtOH’s impact on extinction retrieval. We showed that EtOH increased conditioned responding following fear extinction and interpret this finding as extinction impairment, in line with the abovementioned work. One alternative, but not mutually exclusive, explanation is that EtOH augmented conditioned responding by strengthening the originally conditioned fear memory, thus overcoming the inhibitory extinction memory. This mechanism seems improbable, however, considering that EtOH did not impact freezing performance in response to the original “target” 5-kHz CS (Fig. 1b). EtOH exposure following a relatively “stronger” extinction learning paradigm failed to impact subsequent retrieval performance, however decreased CRs were observed at a remote (20 days) time point. It is possible that EtOH-induced neuroadaptions occurring over time may have degraded or changed the original and/or extinction fear memory, resulting in reduced CRs. How the passage of time, systems consolidation processes, and lingering EtOH-induced neuroadaptations interact with fear memory expression is an open question. A final observation was that the context renewal effect (ABA design) was robust (>80% freezing) and equivalent after either massed or distributed extinction training^97^.

### EtOH and cued fear memory expression

No effects of EtOH were detected on retrieval of the original cued fear memory, a cued fear memory formed using a lower US intensity, retrieval of the original fear memory at a remote time point, or when EtOH was timed 24 hrs following training. These findings do not support the original study hypothesis and converging evidence indicating that chronic EtOH exposure following fear conditioning enhances retrieval^22–24^. In addition to cued fear memory retrieval, EtOH spared hippocampal-dependent memory retrieval, as measured during the pre-CS periods and the extinction context renewal experiments. There is considerable evidence to suggest that EtOH changes hippocampal structure and function^20,98,99^, although there are other studies indicating no effects of EtOH on hippocampal-dependent memory^63^.

### Clinical relevance

PTSD develops in some people (approximately 8% incidence in U.S.) after exposure to a traumatic event and is characterized by intrusive re-experiencing, avoidance, hyperarousal, and negative cognitive/mood symptoms^9^. Of this cluster of symptoms, re-experiencing trauma has been most definitively linked with dysfunction in the neuronal circuits underlying fear learning and memory^100^. One prominent hypothesis posits, that in PTSD, a reduction in top-down control (i.e., medial prefrontal cortex) of excessive reactivation of traumatic fear memories (i.e., basolateral amygdala) contributes to the over re-experiencing of thoughts and mood related to the traumatic event(s)^101^. PTSD and AUD are highly comorbid, with drinking rates among those with PTSD ranging from 30–60%^102,103^. Alcohol drinking tends to increase following trauma exposure^3^ and is associated with worse PTSD symptoms and poorer treatment outcomes^6^, indicating an interaction between excessive alcohol use and the expression of PTSD symptoms. Both PTSD and AUD have been linked with changes in the structure and function of the prefrontal cortex and amygdala^75,104–106^. Here we show in mice that EtOH exposure, following the establishment or extinction of a fear memory, increased fear memory generalization, impaired fear extinction retrieval, and produced neuroadaptations in an IL circuit. With respect to the Research Domain Criteria (RDoC), these data indicate that EtOH-driven changes in IL circuitry may contribute to how acute threat and/or adaptive responses to acute threat is processed^107^. Stimulating IL excitatory projection neurons normalized EtOH-induced overgeneralization. The human homologue of the rodent IL is Brodmann’s area 25^91^. Like the rodent IL, area 25 is thought to exert top-down control over the amygdala, and defects in this circuitry have been suggested to underlie mood disorders and PTSD. This raises the intriguing possibility that deep brain stimulation (DBS) of area 25 in humans could alleviate some PTSD symptoms, particularly in those with symptoms exacerbated by excessive alcohol use. Indeed, DBS of area 25 has already been shown to reduce symptoms of major depressive disorder^108^. Overall, the present data support the hypothesis that excessive alcohol use selectively contributes to impairments in the modulation of fear memory expression (generalization and extinction), via neuroadaptations targeting top-down control, yielding exaggerated fear responses and, leading to poorer clinical outcomes in patients suffering from PTSD.

## Supplementary information

Supplemental Info
