# Supplementary material for: Chemogenetic stimulation of the infralimbic cortex reverses alcohol-induced fear memory overgeneralization
Source: Sci Rep. 2019 Apr 30;9:6730. doi: 10.1038/s41598-019-43159-w (PMC6491487; doi:10.1038/s41598-019-43159-w)
Supplement: Supplementary file 1 — Supplemental Info [file 41598_2019_43159_MOESM1_ESM.pdf]

Supplementary Info

Chemogenetic stimulation of the infralimbic cortex reverses alcohol-induced fear memory overgeneralization

Scarlata MJ, Lee SH, Lee D, Kandigian S, Hiller AJ, Dishart JG, Mintz GE, Wang Z, Coste G, Mousley A, Soler I, Lawson K, Ng AJ, Bezek JL, Bergstrom HC

Vassar College, Department of Psychological Science, Program in Neuroscience and Behavior, Poughkeepsie, NY 12604 USA

Figure S1

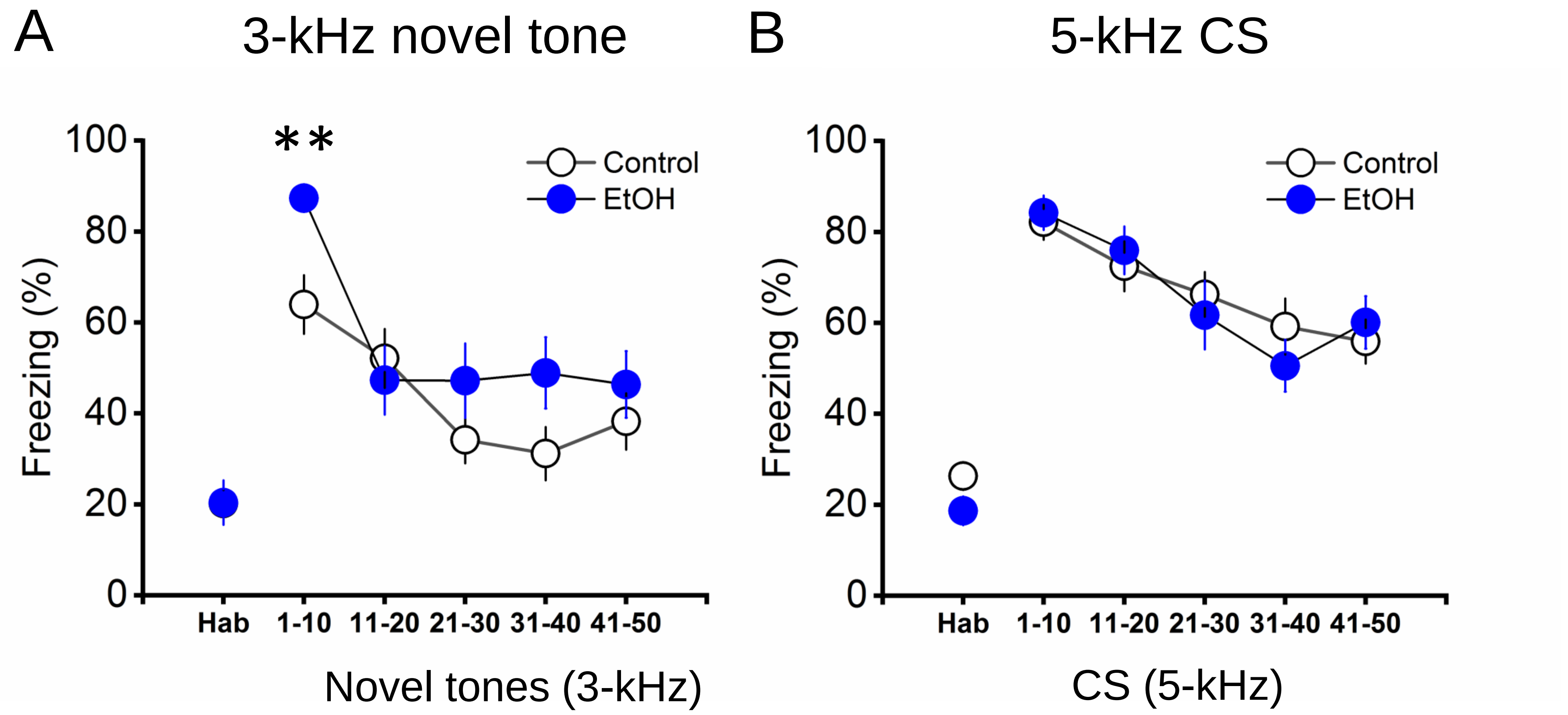

Figure S1: EtOH selectively increased conditioned freezing in response to the novel tone. A) Chronic EtOH increase freezing during presentation of novel tones (3-kHz) 1-10 only. B) There were no effect of EtOH on freezing in response to the original CS. Repeated measures ANOVA followed up with Bonferroni corrected ANOVA. n = 12-15/group, ***p* < .01

Figure S2

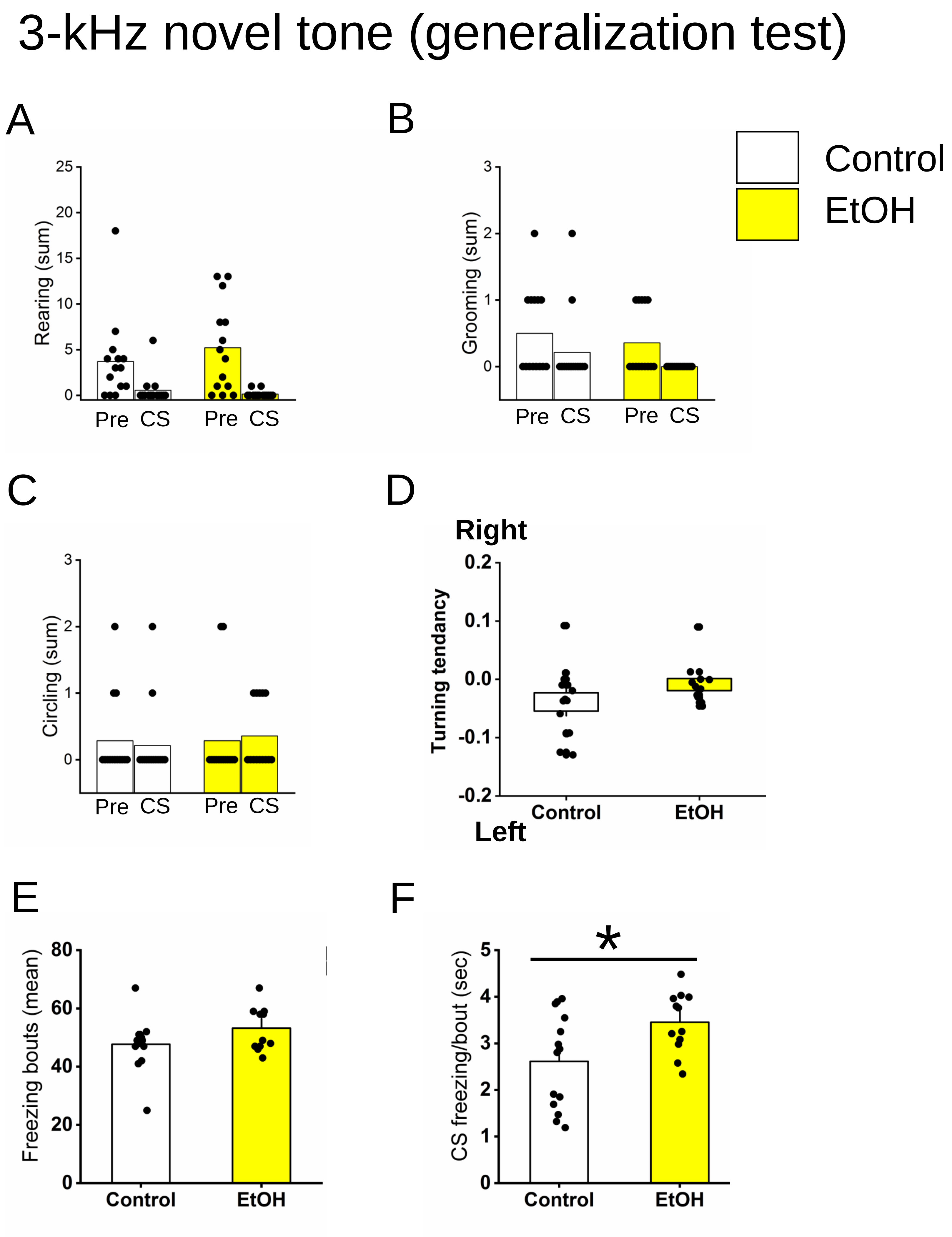

Figure S2: A “multi-measure” analysis of behavior prior to, and during, the presentation of the novel 3-kHz tone (generalization test). A-D) There were no treatment differences in rearing, grooming, circling, or the direction of circling (turning tendency). E) There were no differences in the number of freezing bouts in response to the novel tone. F) Chronic EtOH increased the duration of freezing per freezing bout. Fisher’s exact test (A-D) and one-way ANOVA (E-F). n = 12-15/group, $*p < .05$

Figure S3

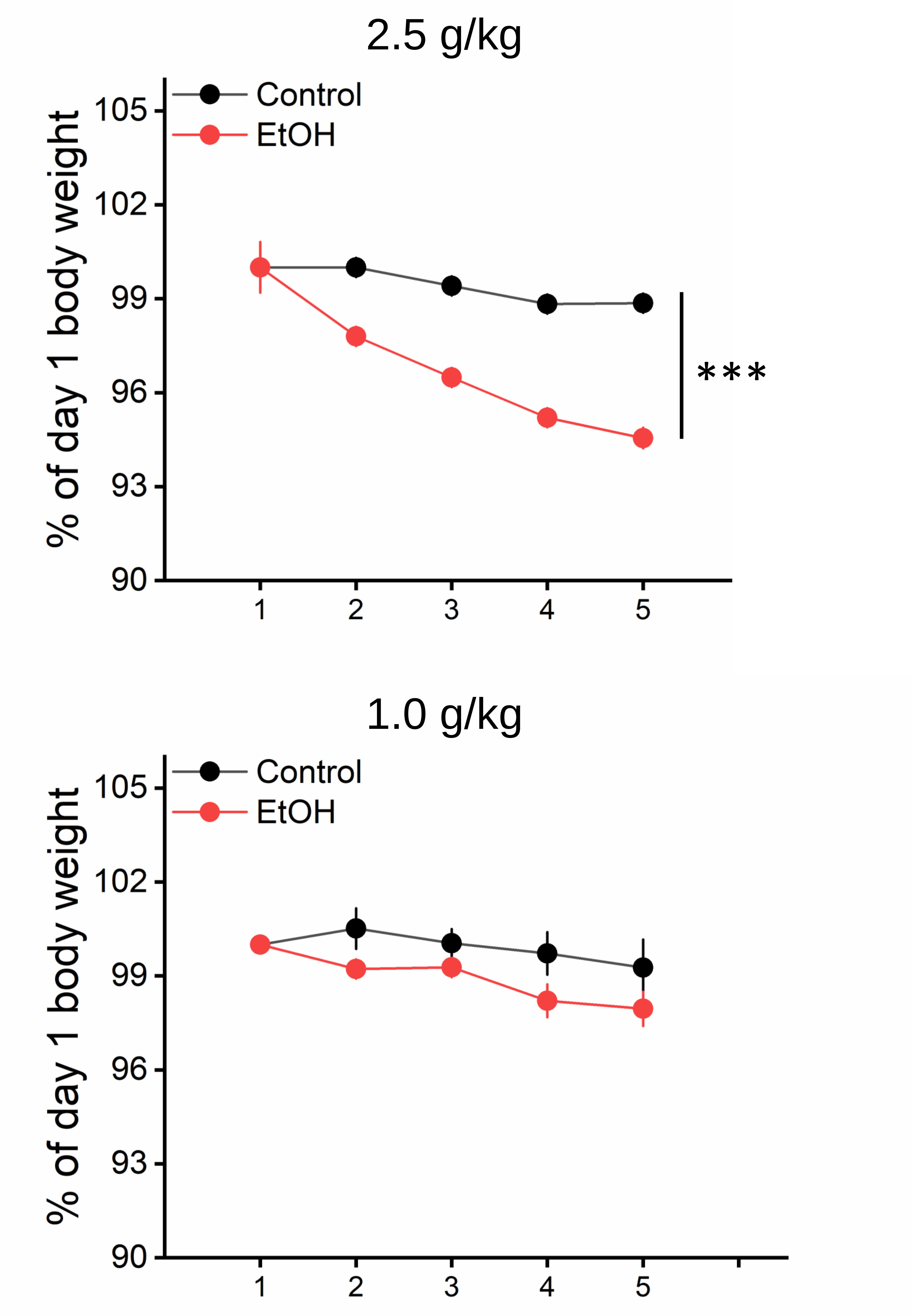

Figure S3. Intraperitoneal EtOH injections (2.5 g/kg) produced body weight loss over the course of the 5-day injection schedule (RMANOVA, F[1, 96] = 98.1; *p* > .001). Weight loss in the EtOH group was evident early, on the second day of dosing (t[96] = 9.8; p > .001). There was no body weight loss in response to the 1.0 g/kg injection. *** *p* < .001

Figure S4

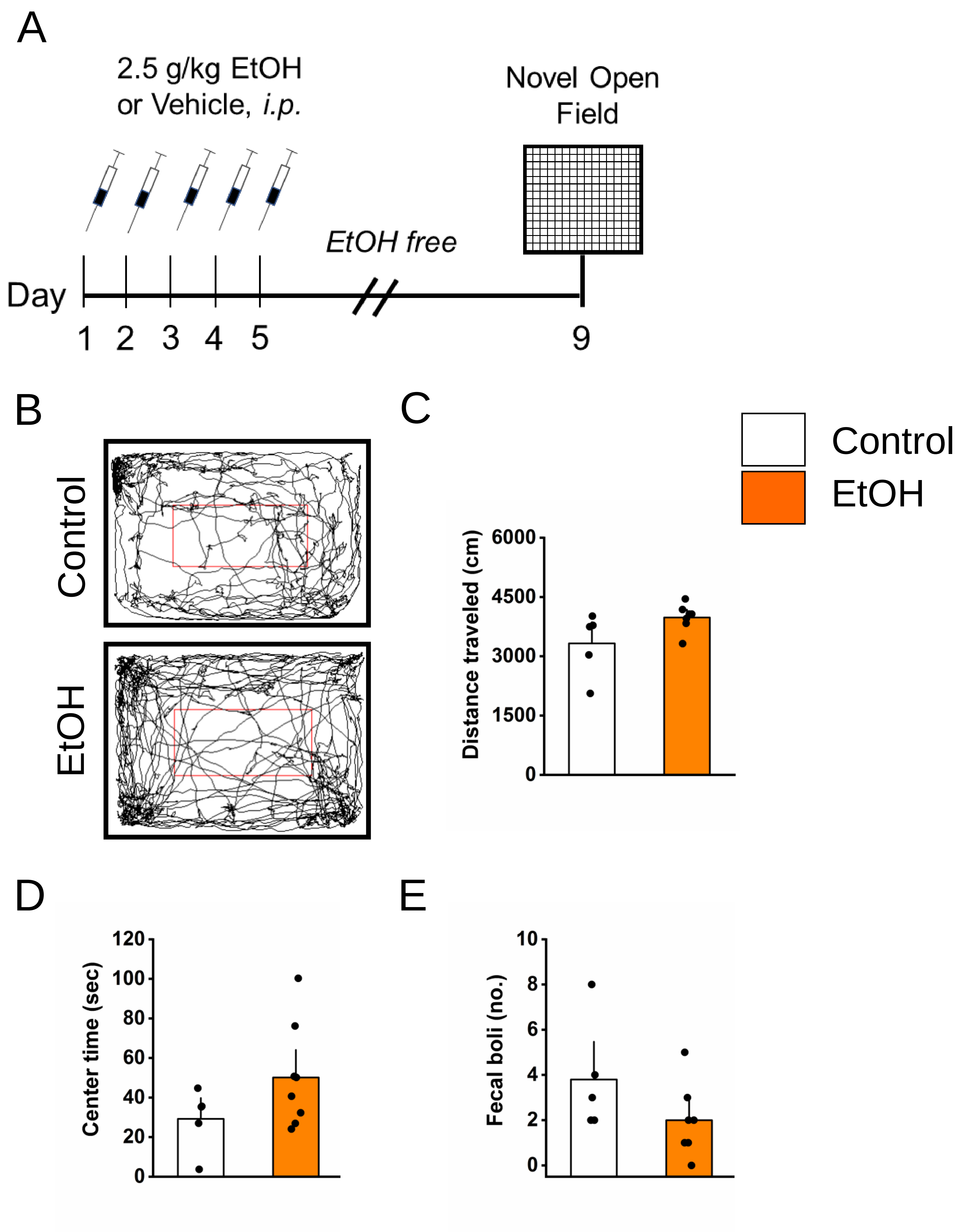

Figure S4: There were no effects of chronic EtOH on behavior in the novel open field. A) Schematic depicting the experimental design for the novel open field test. Importantly, the open field test was conducted on the same day following EtOH exposure as fear retrieval testing. B) Representative path analysis of locomotor behavior in the open field. There were no differences in distance traveled (C), center time (D) or fecal boli counts (E) between the EtOH and Control groups. n=5-7/group

Figure S5

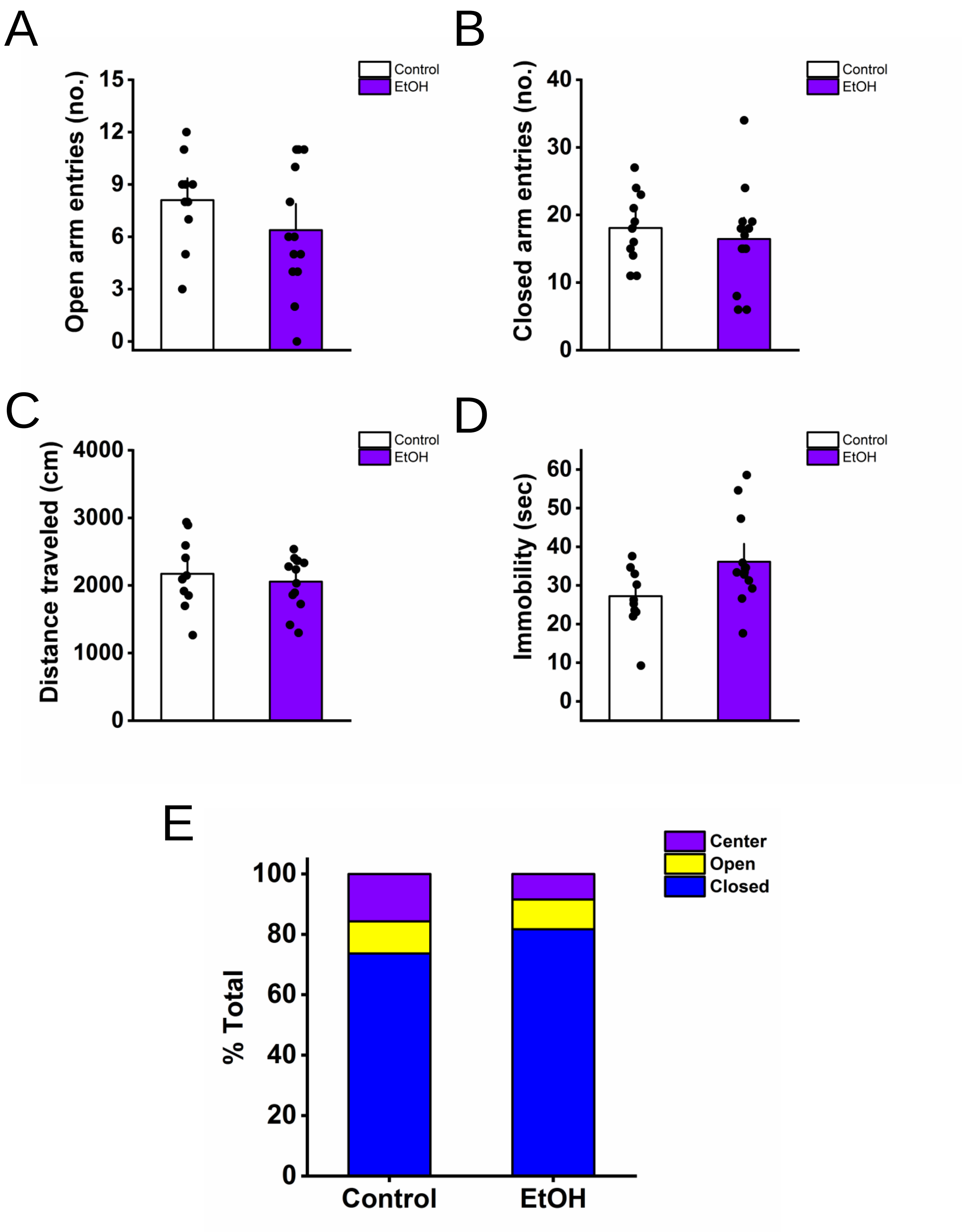

Figure S5: There were no effects of chronic EtOH on behavior in the elevated plus maze. The EPM test was conducted approximately 4 hrs following the generalization test. A-E) There were no treatment differences in open arm entries, closed arm entries, distance traveled, immobility or percentage duration in each arm. one-way ANOVA. n = 11-13/group

Figure S6

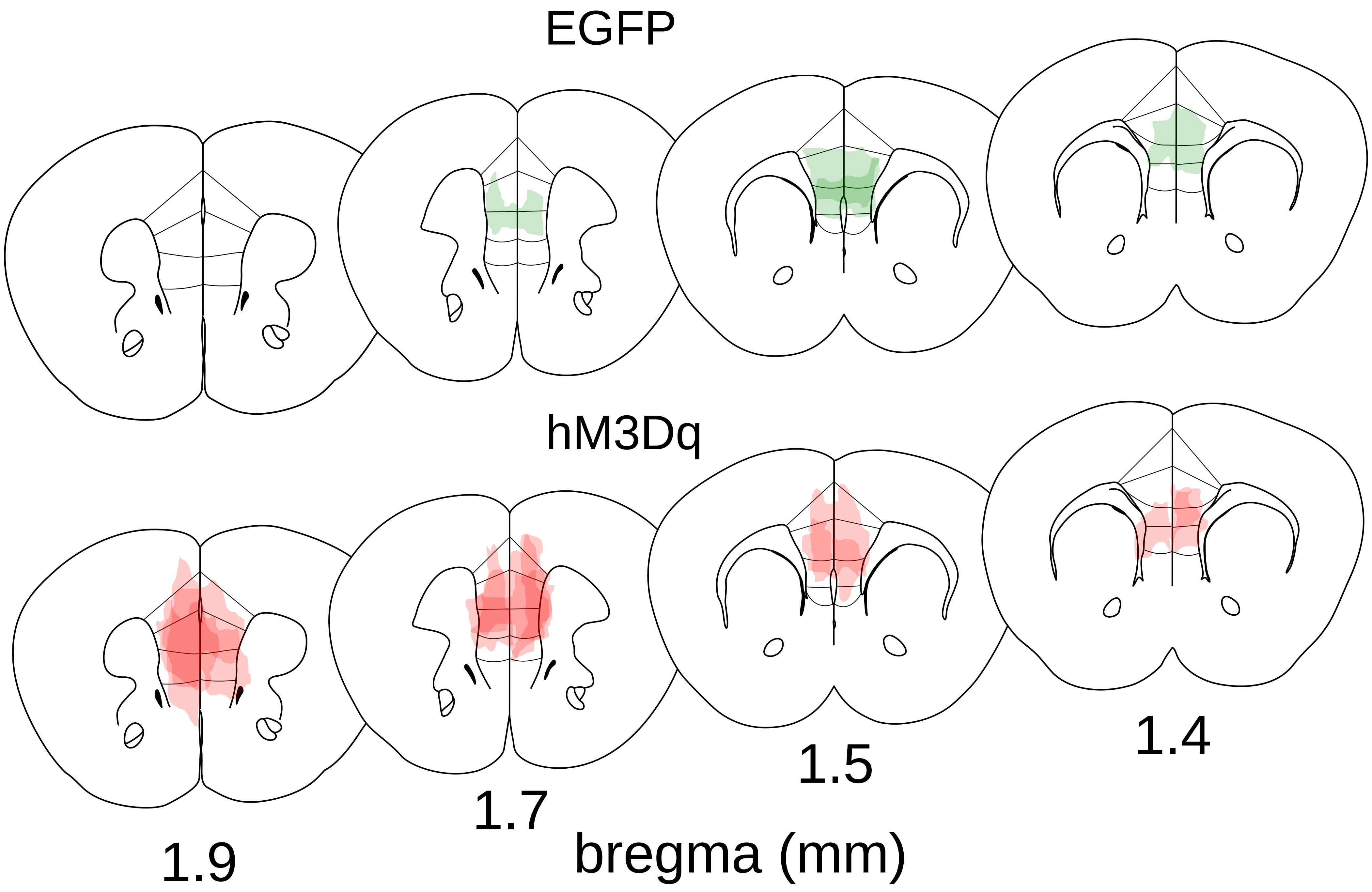

Figure S6: The extent of AAV expression in the mPFC across four coronal planes is depicted. The pattern of AAV expression was digitally reconstructed, shaded (10% opacity), and aligned in a common stereotaxic group space across all mice. Relatively darker shaded regions show greater overlap across individuals. Mouse brain atlas images modified from [43].

Figure S7

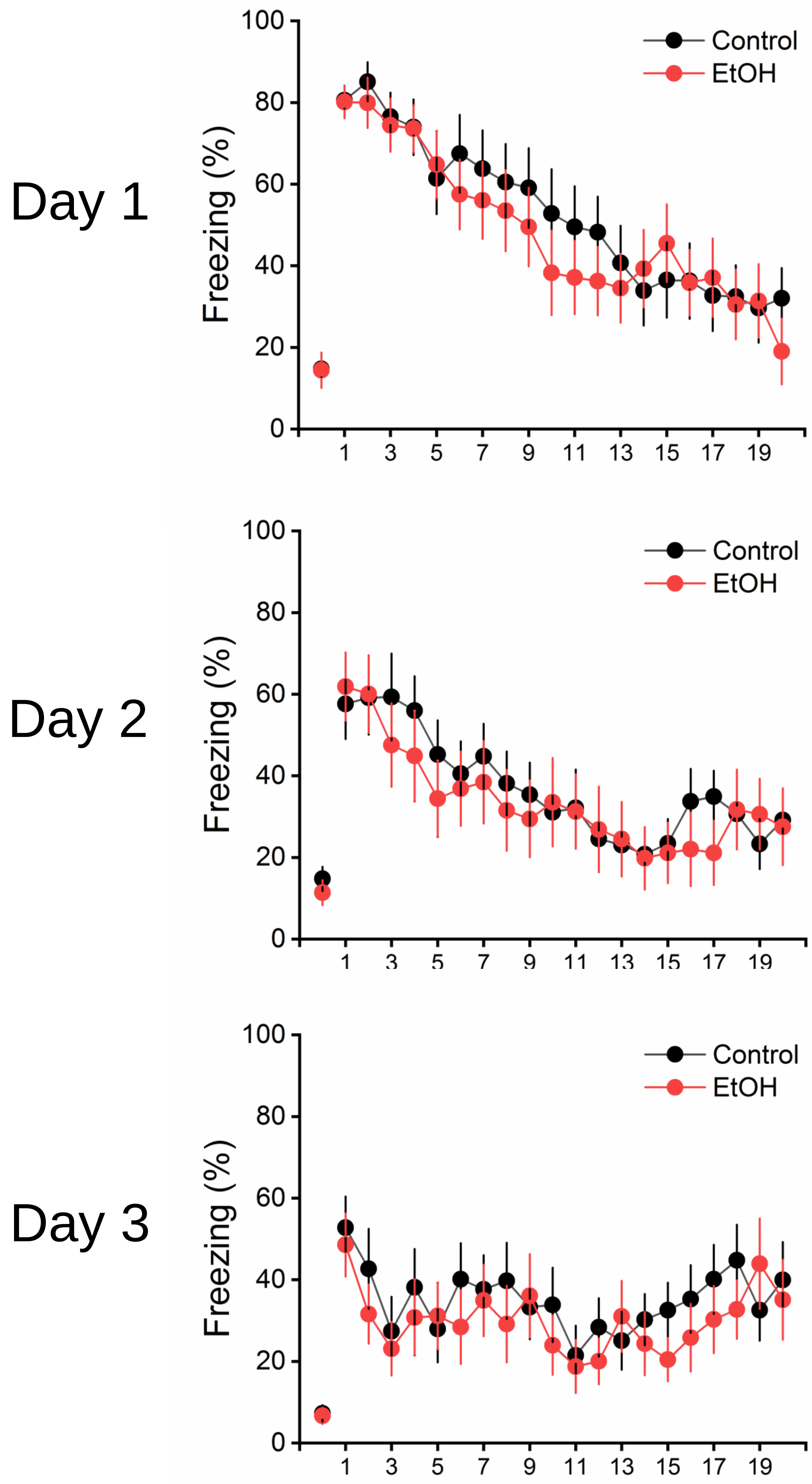

Figure S7: There was a decrease in freezing over 3-days of presentation of the CS (20 CSs on each day). Mixed repeated measures ANOVA. n = 14/group
